# Objective and subjective consequences of pandemic-related study program changes for the perception of the practical year – a comparison of medical students in Germany with and without a second national board examination

**DOI:** 10.3205/zma001635

**Published:** 2023-06-15

**Authors:** Miriam Gisi, Vanessa Ferrari, Felix Dubon, Martin R. Fischer, Matthias Angstwurm, Markus Berndt

**Affiliations:** 1 LMU University Hospital, LMU Munich, Central PJ Coordination, Munich, Germany; 2 LMU University Hospital, LMU Munich, Faculty of Medicine, Office of the Dean of Studies, Munich, Germany; 3 LMU University Hospital, LMU Munich, Institute of Medical Education, Munich, Germany; 4 LMU University Hospital, LMU Munich, Medical Clinic and Polyclinic IV, Munich, Germany

**Keywords:** practical year, national board examination, COVID-19, career, stress

## Abstract

**Background:**

Due to SARS-CoV-2, the Bavarian Ministry of Health decided in April 2020 to postpone the second national board examination in human medicine and to bring forward the start of the practical year (in German: Praktisches Jahr, further abbreviated with PJ) from May to April 2020. The different tertial times made it necessary for affected students to reorganise the PJ and rendered the preparation for the national board examination that had already taken place obsolete. As a result, students had to prepare for it again after the PJ and take it together with the third national board examination.

**Research question:**

How do students affected by the early PJ differ in their perception of the practical year and in their psychological well-being from the comparison groups with a regular PJ schedule?

**Methodology:**

The study is based on quantitative data from the Dean of Studies Office of the Medical Faculty of Ludwig-Maximilians-Universität München (LMU) and an online survey. The sample consists of LMU students who started the early PJ in April 2020 (*n*=86) and two comparison groups: The cohort of LMU students who started their PJ regularly in May 2019 (*n*=50), and students from other German universities who started their PJ regularly in May 2020 (*n*=98) and took the second national board examination in human medicine in spring 2020.

**Results:**

For students affected by the early PJ, there were measurable negative effects on the choice of training institutions, the quality of the PJ content, preparation for the national board examinations, and career planning. Compared to regular students from other federal states, affected students reported higher psychological stress, with comparable resilience.

**Conclusion:**

It can be assumed that the insights gained apply to the entirety of medical students in the affected federal states of Bavaria and Baden-Württemberg. As a conclusion, we make the recommendation to include the position of the students in decisions of great consequence.

## Zusammenfassung

**Hintergrund:**

Aufgrund von SARS-CoV-2 hat das Bayerische Gesundheitsministerium im April 2020 entschieden das zweite Staatsexamen Humanmedizin zu verschieben und den Beginn des Praktischen Jahres (PJ) von Mai auf April 2020 vorzuverlegen. Die abweichenden Tertialzeiten machten für betroffene Studierende eine Reorganisation des PJs notwendig und die bereits erfolgte Vorbereitung auf das zweite Staatsexamen hinfällig. Dieses musste in der Folge außerplanmäßig zeitgleich mit dem dritten Staatsexamen nach dem PJ erneut vorbereitet und absolviert werden.

**Fragestellung:**

Wie unterscheiden sich von der PJ-Verschiebung betroffene Studierende in ihrer Wahrnehmung des Praktischen Jahres und in ihrem psychischen Wohlbefinden von den Vergleichsgruppen mit regulärem PJ-Ablauf?

**Methodik:**

Die Studie basiert auf quantitativen Daten des Studiendekanats der Medizinischen Fakultät der Ludwig-Maximilians-Universität München (LMU) und einer Online-Umfrage. Die Stichprobe besteht aus Studierenden der LMU, die das vorgezogene PJ im April 2020 begannen (*n*=86) sowie zwei Vergleichsgruppen: Die Kohorte Studierender der LMU, die im Mai 2019 regulär ihr PJ begannen (*n*=50), und Studierende anderer deutscher Universitäten, die das PJ im Mai 2020 regulär antraten (*n*=98) und das zweite Staatsexamen Humanmedizin im Frühjahr 2020 absolvierten.

**Ergebnisse:**

Für von der Verschiebung betroffene Studierende zeigten sich messbare negative Auswirkungen auf die Wahl der Ausbildungseinrichtungen, die Qualität der PJ-Inhalte, die Vorbereitung auf die Staatsexamina und die Karriereplanung. Dabei berichteten betroffene Studierende im Vergleich zu regulären Studierenden anderer Bundesländer eine höhere psychische Belastung, bei vergleichbarer Resilienz.

**Schlussfolgerung:**

Es kann davon ausgegangen werden, dass die gewonnenen Einsichten für die Gesamtheit der Medizinstudierenden in den betroffenen Bundesländern Bayern und Baden-Württemberg zutreffen. Als Schlussfolgerung sprechen wir die Empfehlung aus, die Position der Studierenden in Entscheidungen von großer Tragweite miteinzubeziehen.

## 1. Introduction

### 1.1. Background

In the ordinance on the deviation from the licensing regulations for physicians in the event of an epidemic situation of national significance of April 1st, 2020, the Federal Ministry of Health recommended the postponement of the second stage of the national board examination for physicians (further called second national board examination or M2) and to bring forward the practical year (in German: Praktisches Jahr, further abbreviated with PJ) due to the spread of Sars-CoV-2. This was done in view of the risk of infection during the exam and the need for support in the health system. The Ministry left it up to the federal states to decide whether to deviate from this rule [https://www.gesetze-im-internet.de/epi_appro2002abwv/index.html]. The state examination boards in Baden-Württemberg and Bavaria were the only federal states to decide to follow the recommendation of the Federal Ministry of Health and brought forward the start of the PJ from the original date of 18.05.2020 to 20.04.2020. This resulted in a shortening of each tertial to 15 weeks [1].

The students in Bavaria and Baden-Württemberg affected by this decision had to reorganise existing PJ plans into a shortened PJ at short notice due to the deviating tertial times. Their already allocated examination date for the second national board examination was cancelled at short notice and was postponed until the end of their studies. This happened after the learning phase (usually several months) had already taken place. As a result, affected students had to prepare again for the M2 after the PJ and at the same time for the third national board examination (M3). In terms of the procedure, this is similar to the so-called Hammerexamen according to the licensing regulations for physicians of 2002, but without the corresponding conception of the curriculum and the learning phases in the run-up, which was common at that time. In contrast, the students for whom the M2 and the PJ start took place regularly (rPJ-20) were able to take the second national board examination before the PJ and start it as planned. At the end of the PJ, they had to prepare exclusively for the third national board examination.

The decision to cancel the second national board examination was strongly criticised by the German Medical Students’ Association (in German: Bundesvertretung der Medizinstudierenden in Deutschland e.V.) due to possible disadvantages for the affected students [2]. Particular emphasis was placed on the different conditions between medical students affected by the early PJ and those who wrote the M2 regularly and started the PJ regularly, as well as the shortened phase of exam preparation for students with an early PJ (referred to as vPJ-20 in this article).

It is well studied that clinical clerkships and practical experience strongly influence both the career planning of medical students and their professional orientation [3], [4], [5], [6], [7]. The PJ in particular offers the opportunity to gain experience with future employers [8]. Good career planning is therefore related to the possibility of being able to choose specialties and clinics for the individual tertials and to ensure good learning conditions [9]. In this context, both the choice of PJ training institutions and mobility during the PJ (within Germany and abroad) are crucial. 

In medical education, psychological well-being, which can be influenced by stressful situations and test anxiety, is of particular importance [10], [11], [12], [13], [14], [15], [16]. Psychological impairments are more frequent among university students than in the general population [17]. Therefore, they are considered a vulnerable social group [18]. Medical students in particular are exposed to high levels of stress, so that even students who begin their studies with good mental health often suffer from depression and anxiety in the course of their studies [19], [20].

### 1.2. Research question

The present study explored how the conditions outlined above affected medical students. The focus was specifically on the following overarching questions.

1. How do students affected by the early PJ differ in their career planning and in their perception of the practical year from the comparison groups with regular PJ schedules?
2. How do students affected by the early PJ differ in their self-assessment of psychological well-being from the comparison groups with regular PJ schedules?

## 2. Methodology

### 2.1. Sample

The study was based on an explorative qualitative and quantitative analysis of anonymised data from the Dean of Studies Office of the Medical Faculty of the Ludwig-Maximilians-Universität München (LMU) and on data from an anonymous survey. The target group were the 227 students who had registered for the M2 in 2020 and thus were affected by the postponement. Of these, 185 (81.5%) started the early PJ (vPJ-20), and 42 (18.5%) did not start the PJ for various reasons. 86 out of 185 students participated in the survey.

The comparison groups were 50 PJ students from LMU who had completed their PJ regularly from May 2019 (rPJ-19) and 98 PJ students from other German universities whose M2 and PJ start had taken place regularly (rPJ-20), see table 1 (Tab. 1). The rPJ-20 group was made up of students from the universities of Aachen, Göttingen, Hamburg, Hannover, Kiel, and Münster. Students of vPJ-20 and rPJ-19 took part in the online survey in July/August 2020, students of rPJ-20 in October 2020.

### 2.2. Measuring instrument

In the online survey, the participants were asked about the organisation and planning of the PJ as well as the reasons for their choice of hospital (6 items) and planning of their future career (3 items). The students gave self-assessments on stress (3 items), resilience (4 items) and exam anxiety (2 items). The resilience items are based on the resilience scale by Schumacher et al. [21]. Due to the length of the survey, only some of the items were taken from this scale. The evaluation was carried out by summing and averaging the raw item values, whereby a high score stands for high resilience (internal consistency based on our sample: Cronbach’s α=.75). All other items are self-generated by the authors in collaboration with experts (PJ officer and dean of studies). Specific questions about the pandemic were asked at the end (7 items). Most of the items were answered on a 6-point Likert or dichotomous scale. For some questions, students had the opportunity to add free text (see attachment 1 ).

In the descriptive analysis, questionnaires that were not fully completed were also taken into account. Inferential statistical analyses were carried out with the Statistical Package for the Social Sciences (SPSS) version 26 (IBM). If the prerequisites were fulfilled, possible effects were examined using ANOVA for variance analysis, and differences between the groups were also examined with pairwise comparisons.

## 3. Results

### 3.1. Influence of the M2 postponement and early PJ on students’ career planning and perception of the PJ

#### 3.1.1. Completion of the PJ tertials

Good career planning includes organising one's PJ with full choice of PJ training institution and planning mobility. The students indicated that the future career (specialist training) was an important criterion for the choice of institution. 38.3% in the vPJ-20 group believe that the PJ can influence their choice of specialisation. 58.1% of them already know in the first tertial that they want to apply to their PJ position. Furthermore, the students believed that they would have worse chances of applying without the grade from the M2, as they believe that applications for a position take place during the PJ with the grade from the M2. This data is supplemented qualitatively in isolated cases; as an example, one person formulated this as follows: “There is no longer a national board exam. In the inter- and national competition, the students from Bavaria and Baden-Württemberg have suffered an extreme setback. None of us can now apply for a doctor’s position in time with an M2 [certificate], which is commonplace as a medical student. [...]”.

The elective subject was planned independently of the pandemic or the postponement. All groups were able to complete the majority of the tertials in the desired elective subject, but sometimes in a different teaching hospital than planned. Almost twice as many students in group rPJ-19 spent a tertial abroad compared to students in groups vPJ-20 and rPJ-20. A significantly higher proportion of students in groups vPJ-20 and rPJ-20 had to cancel their tertial planned abroad (see table 2 (Tab. 2)).

36% of the students in vPJ-20 stated that they had rescheduled their PJ due to the limited mobility abroad. According to 31% of the students, this rescheduling was due to the change of the start dates and the restriction of inland mobility (due to the deviating tertials). 12% of the respondents gave both reasons. 

In terms of the tertials actually taken, the proportion of external tertials (within and outside Germany) decreased in general surgery compared to the previous year (-11.2%), while in internal medicine and elective subjects there were hardly any shifts in external tertials compared to the previous year. All three tertials in the vPJ-20 group were increasingly planned in LMU teaching hospitals (see table 3 (Tab. 3)).

A total of 53% of the externally planned vPJ-20 tertials within Germany could not take place as planned, as already organised PJ positions in Germany were cancelled or reset by hospitals due to the changed start times of vPJ-20. As a result, tertials originally planned for vPJ-20 could not take place and the PJ had to be reorganised in a very short time. This data is supplemented in isolated cases by free-text comments that reveal that only very committed students were able to book a new place outside their home university with considerable organisational effort. As an example, one person describes this as follows: “Because of the COVID-19 regulation, the possibility of completing two of three tertials in NRW only arose at very short notice and after much email correspondence and many telephone calls. The third tertial is still up in the air”. 

A total of 94.6% of the vPJ-20 group disagree with the postponement of the M2. Of these, 60.4% had considered not taking the PJ; at 18.5%, this consequence was drawn by more than twice as many M2 candidates as in the previous year. In fact, 42 out of 227 students (18.5%) who had registered at LMU for the second national board examination in April 2020 did not take the PJ. Of these 42 students, 85% stated that they had not started the PJ explicitly due to the changed dates of the PJ and the M2 by the Bavarian State Examination Office. In May 2019, 233 students had registered for the M2 and 21 had decided not to start the PJ in May 2019 (9.0%). This corresponds to an increase of over 100%.

#### 3.1.2. PJ procedure 

**Figure 1:**
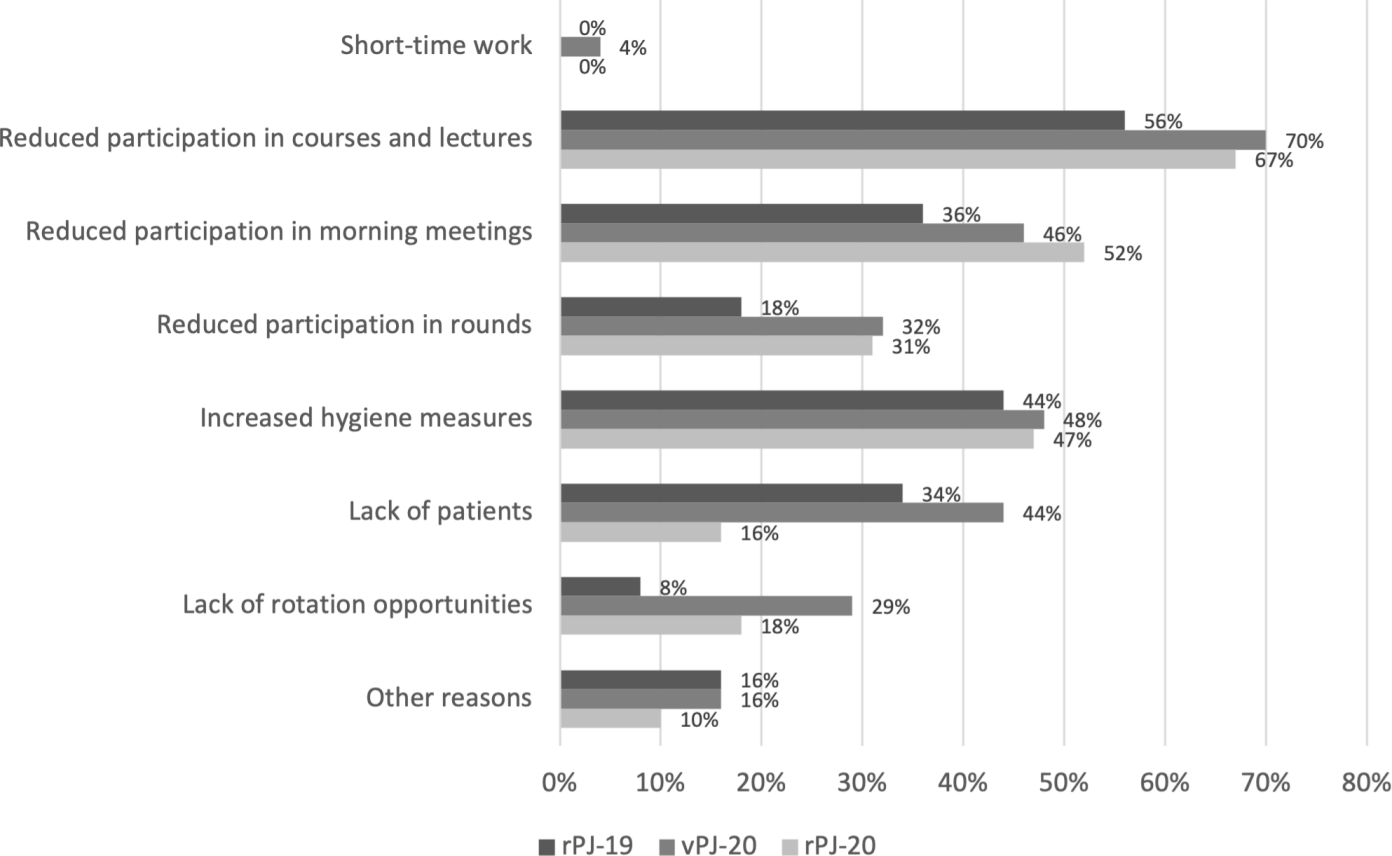
Responses to the item: “What restricted the regular PJ procedure on your ward?”

The quality of the PJ training influences the achievement of higher-level and subject-specific learning goals as well as the possibility to pursue the desired career. Although only 9.3% of the respondents were directly affected by the pandemic (quarantine, illness), 65% of the students in vPJ-20 reported not having experienced a regular PJ. The limitations in the PJ process were reported as significantly greater in vPJ-20 than in rPJ-19 and rPJ-20, *F*(2,227)=22.30, *p*<.001, partial η2=.16 (*M*=2.88, *SD*=0.81 vs *M*=2.19, *SD*=0.73 and *M*=2.25, *SD*=0.60, 4-point Likert scale). Here, the lack of patient contact in particular was stated as a limitation (see figure 1 (Fig. 1)).

Other mentioned factors included increased auxiliary work (such as taking swabs, answering the phone etc.), illness in the team, the frequent change of supervisors, the cancellation of lectures and training courses as well as operations, and the poorly proportioned PJ student/patient ratio. The students in vPJ-20 rated their ability to sufficiently often participate in learning opportunities significantly lower than in rPJ-19 and rPJ-20, *F*(2,230)=14.02, *p*<.001, partial η2=.11 (*M*=2.40, *SD*=1.56 vs *M*=3.60, *SD*=1.59 and *M*=3.52, *SD*=1.63, 6-point Likert scale). Overall, they felt less prepared for their national board examinations than both comparison groups *F*(2,230)=10.62, *p*<.001, partial η2=.09, although this could not be confirmed by an analysis after all M2 results were available. Thus, there was no significant difference in grades between the vPJ-20 and the rPJ-20 students (*p*=.21), and the grades of the vPJ-20 group were on average comparable to the rPJ-19 group (grade 2.94 vs. 3.03).

### 3.2. Influence of the M2 postponement and early PJ on students’ psychological well-being

**Figure 2:**
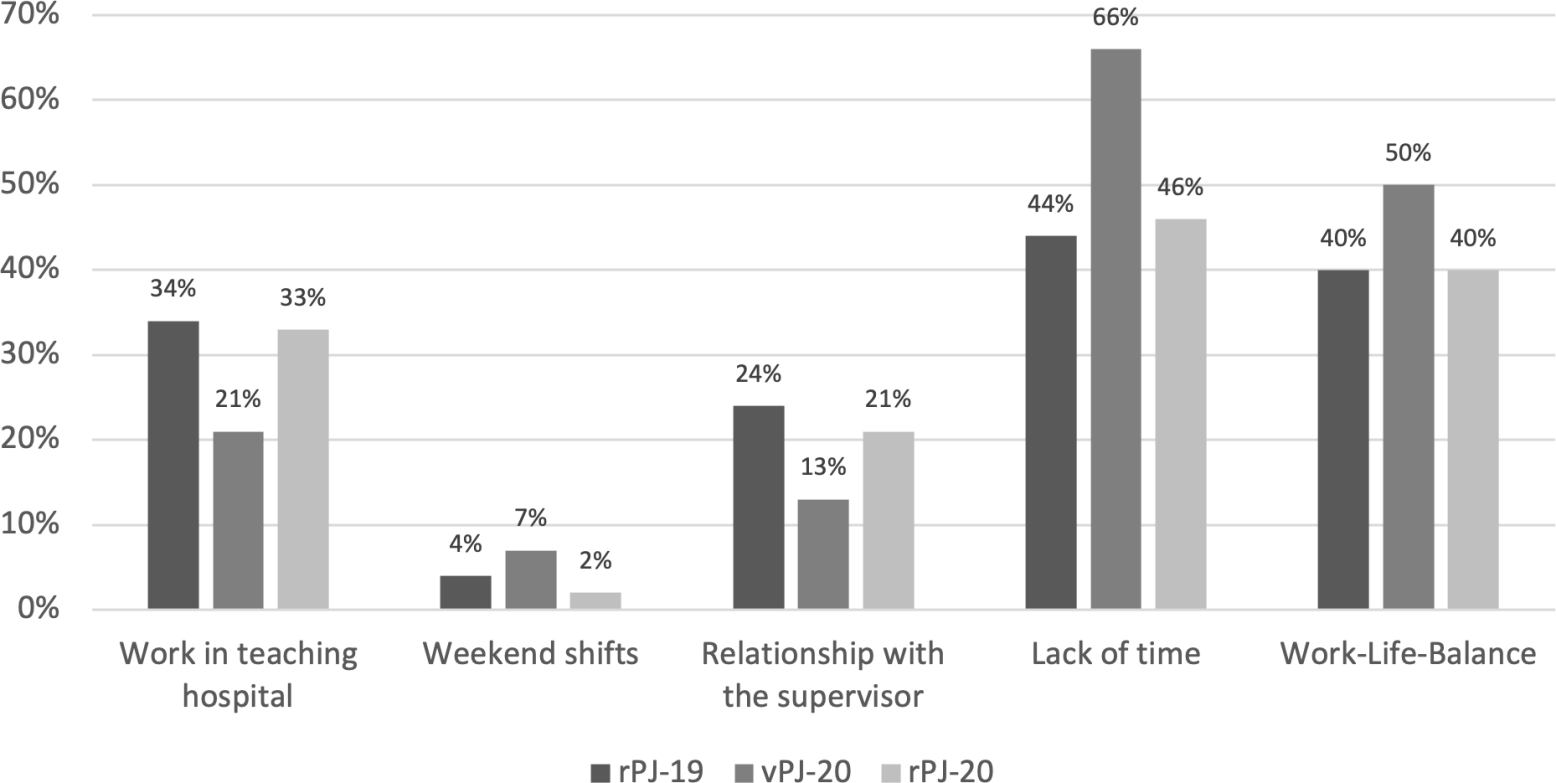
Response to the item: “Which aspects of the activities in the PJ were particularly stressful for you?”

There was no significant effect between the groups in the self-assessment of the ability to cope with stress, *F*(2,229)=2.56, *p*=.08, n.s. However, post-hoc analyses showed that the group vPJ-20 assessed themselves significantly higher than the group rPJ-20, *p*=.04 with an overall high assessment in each of the three groups. No significant effect was found in resilience *F*(2,229)=2.81, *p*=.06, n.s. Post-hoc analyses showed a significantly higher resilience score in group vPJ-20 compared to group rPJ-20, *p*=.02. In the assessment of the perceived stress level in the PJ, a significant effect could be seen *F*(2,229)=3.13, *p*=.046, partial η2=.03, with pairwise comparisons showing a significant difference only between the two groups rPJ-19 and vPJ-20, *p*=.014. This indicates that the external stressors caused by the postponement of the examination and early PJ further increased the stress level in vPJ-20. Among the stressful aspects, lack of time and work-life balance were highlighted by the vPJ-20 group (see figure 2 (Fig. 2)).

No significant effect was found on test anxiety, *F*(2,228)=2.15, *p*=.12, n.s., but post-hoc analyses showed significantly higher pre-M3 test anxiety in group vPJ-20 compared to group rPJ-20, *p*=.04. Since the average M3 scores in these two groups have historically always differed, a comparison is not informative (average M3 score rPJ-20=1.6). More interesting, however, is the comparison of the M3 results of the groups vPJ-20 (average score 1.83) and rPJ-19 (average score 1.6). However, a direct comparison is not possible, as the exact student composition is not known for these average scores. Furthermore, it is not possible to say which other factors influenced these values.

The level of stress among students in vPJ-20 was partly exacerbated by the feeling of not being heard. This is perceived as a disadvantage by the vast majority of vPJ-20 (86%). Although no quantitative data is available on this, the free-text comments provide some indication to support this assumption. The authors point out that these are partly individual mentions that are not sufficient for a thematic analysis. Some students are convinced that it would have been possible to conduct the national board examination while observing certain hygiene rules. A nationwide solution was desired. The postponement of the national board examination is increasingly described as “unfair”. The psychological strain caused by the long uncertainty and the cancellation at short notice before the M2 was frequently mentioned. In addition, the early assignment to the clinics was seen as “superfluous” and “unnecessary”. For some of the respondents, it was incomprehensible being exposed to the risk of infection in the clinics after the risk of infection was mentioned as a reason for postponing the M2. Specific other stress factors were described that had arisen due to the short-term postponement and had not been considered, such as cancellations of accommodation, organisation of part-time jobs, doctoral theses, and childcare.

## 4. Discussion

### 4.1. Career planning and PJ perception

Compared to the other cohorts, the students in vPJ-20 feared negative effects on their careers, partly because of the pandemic per se, partly because of the early PJ. This perception is based on concrete data (restriction of mobility, activities completed in the first months of the pandemic, reduced learning time, rescheduling of tertials due to changed start times and changed application opportunities) but also on students’ assumptions and fears (fear of lower grades in M2, conviction of having been offered fewer learning opportunities).

It remains interesting that the majority of students seems to believe that this presumed disadvantage is not an unavoidable consequence of the pandemic, but that it was caused or intensified by the postponement of the M2 and early PJ in Bavaria and Baden-Württemberg. The restrictions on free tertial selection were higher after the earlier tertial start in vPJ-20. The assessment of students in the vPJ-20 group that they were significantly less likely to have the opportunity to participate in learning opportunities cannot be substantiated by data.

### 4.2. Psychological well-being

The data on psychological stress indeed show an increased level of stress among students in vPJ-20 compared to rPJ-19, although there is no difference in resilience, i.e. stress coping ability, between rPJ-19 and vPJ-20. In other words, students in vPJ-20 were more stressed than students the year before, with the same resilience. This could be an indication that the COVID-19 pandemic itself led to an increased stress load among students in the PJ – a result supported by other studies [22], [23], [24], [25]. It should be borne in mind that the students affected by the earlier PJ tended to report higher stress levels than the students in the rPJ-20 under the same pandemic condition. The higher stress level in the vPJ-20 group must additionally be evaluated from the point of view that this group rated itself as significantly more resilient than the rPJ-20 group and that the earlier PJ was explicitly cited as one of the reasons.

In the perception of the students, their concerns were not sufficiently heard and taken into account by the political decision-makers in the discussion about the M2 postponement and early PJ. In addition, the students in the vPJ-20 group feared lower grades in the M2, as they had to prepare for it with a shorter learning period. Normally, students are guided by the 100- or 120-day learning plan of well-known learning platforms. This can usually be implemented parallel to the last clinical semester. Parallel to the PJ, learning is not possible to a similar extent. Even if all days of absence are placed at the end of the PJ, the vPJ-20 group has at most 6 weeks of preparation time for the M2 [26]. They have to study for the M2 (13-15 April 2021) and the M3 (May-June 2021) almost simultaneously. This is perceived as a disadvantage by the students. Students in group vPJ-20 reported significantly higher exam anxiety before the M3 compared to students in other federal states (rPJ-20). An actual deterioration of the group vPJ-20 compared to the previous year's group rPJ-19 cannot be shown without knowledge of the exact student composition and the influencing factors. Only the existence of descriptive differences in the published M3 average grades can be noted here. Whether there are further, long-term effects on careers must be investigated in follow-up studies.

### 4.3. Limitations 

When collecting the data, it should be taken into account that the students were surveyed at slightly different times, the rPJ-20 group in October 2020 and the vPJ-20 and rPJ-19 groups in July/August 2020. The reason for this was the difficulty in reaching students from other faculties. The vPJ-20 group was therefore somewhat more affected by the impact of the start of the pandemic and the new circumstances than the rPJ-20 group. In autumn 2020, some of the PJ procedures and teaching could take place more regularly, since the first pandemic wave with complete lockdown was over. In addition, not all medical faculties participated in the study. The study therefore does not give a complete picture of the German university landscape. Nevertheless, the random selection of universities participating in the study is representative for the entirety of medical faculties and their students and includes small and large faculties.

Finally, the surveys are based on self-assessments. In the vPJ-20 group, about 50% of the students participated in the survey, which is more than in rPJ-19. This shows the high relevance of the survey among acutely affected students and the desire to be heard. The high participation in rPJ-20 also indicates that the COVID-19 pandemic is on the minds of PJ students. Nevertheless, the study participants cannot represent the opinion of all students. It is obvious that the pandemic also had an influence on the self-assessments given. For this reason, the explicit naming of other reasons – not related to the pandemic per se – is particularly important, but also the fact that one of the comparison groups also acted under pandemic conditions. 

## 5. Conclusions

The results of the study show that the postponement of the M2 and the early PJ start at the beginning of the COVID-19 pandemic were perceived negatively by the affected medical students in terms of their career planning and psychological well-being. PJ students criticise inequality in terms of their career plans and possibly career opportunities, as well as poorer conditions in the hospitals where the PJ was completed. Although these concerns can be partially refuted by data, the impact on students’ health, particularly in terms of stress levels and exam anxiety, persists. This should be considered by policy makers and potential employers for future decisions. Student responses indicate a desire to be involved. Improved communication between policy makers and students should be sought for future decisions, especially for those with high implications. One possibility for this would be a formalised procedure in which the executive boards of the German Medical Students’ Association and/or the student bodies (in German: Fachschaften Medizin) are explicitly invited to discuss decision options.

## First authorship

The authors Miriam Gisi and Vanessa Ferrari share the first authorship.

## Competing interests

The authors declare that they have no competing interests. 

## Supplementary Material

Questionnaire PJ

## Figures and Tables

**Table 1:**
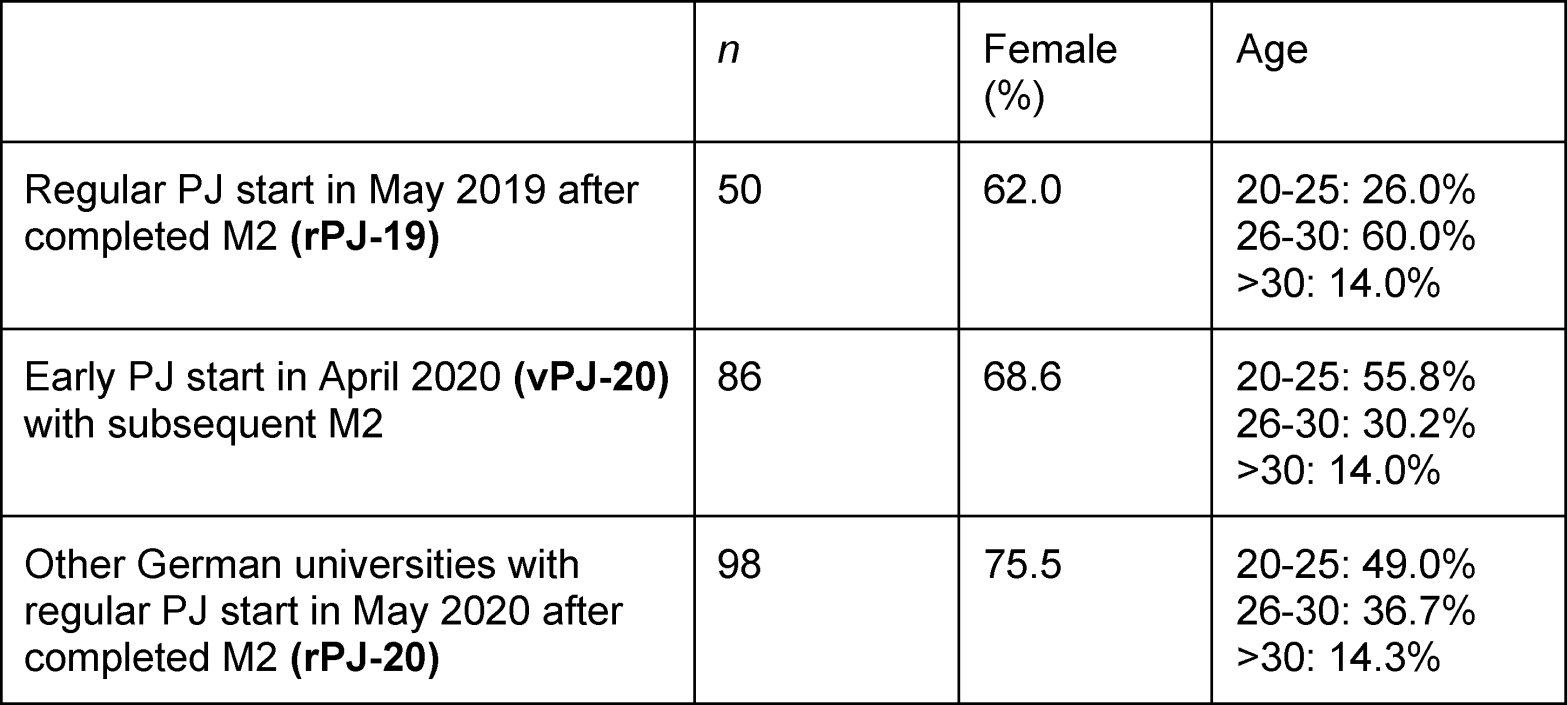
Characteristics of study participants

**Table 2:**
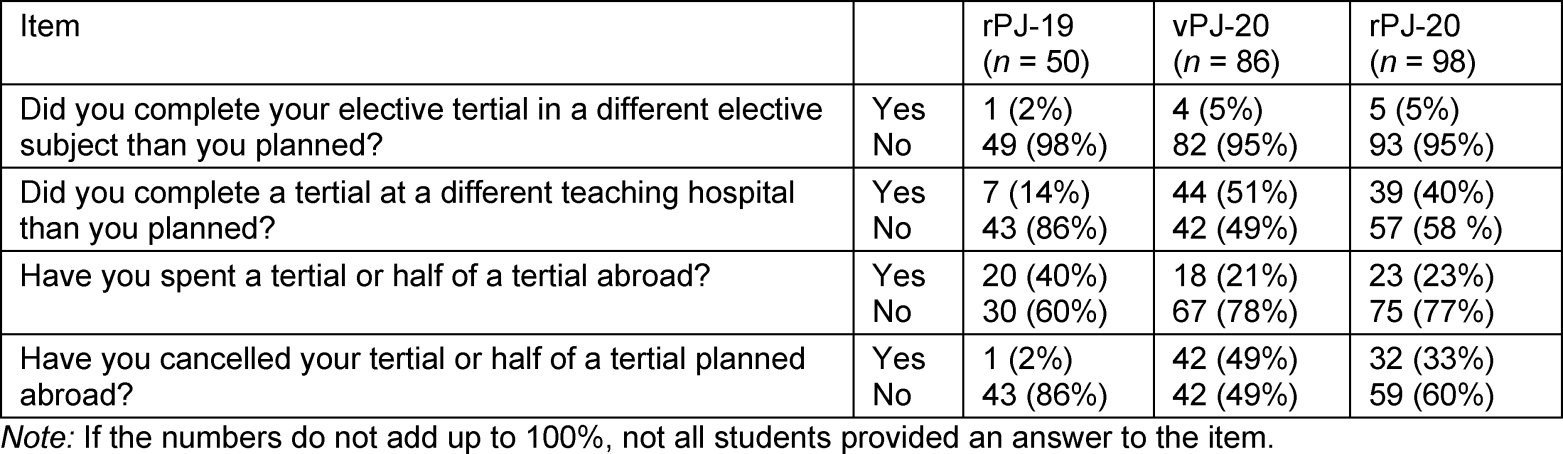
Responses of participants regarding the PJ procedure

**Table 3:**
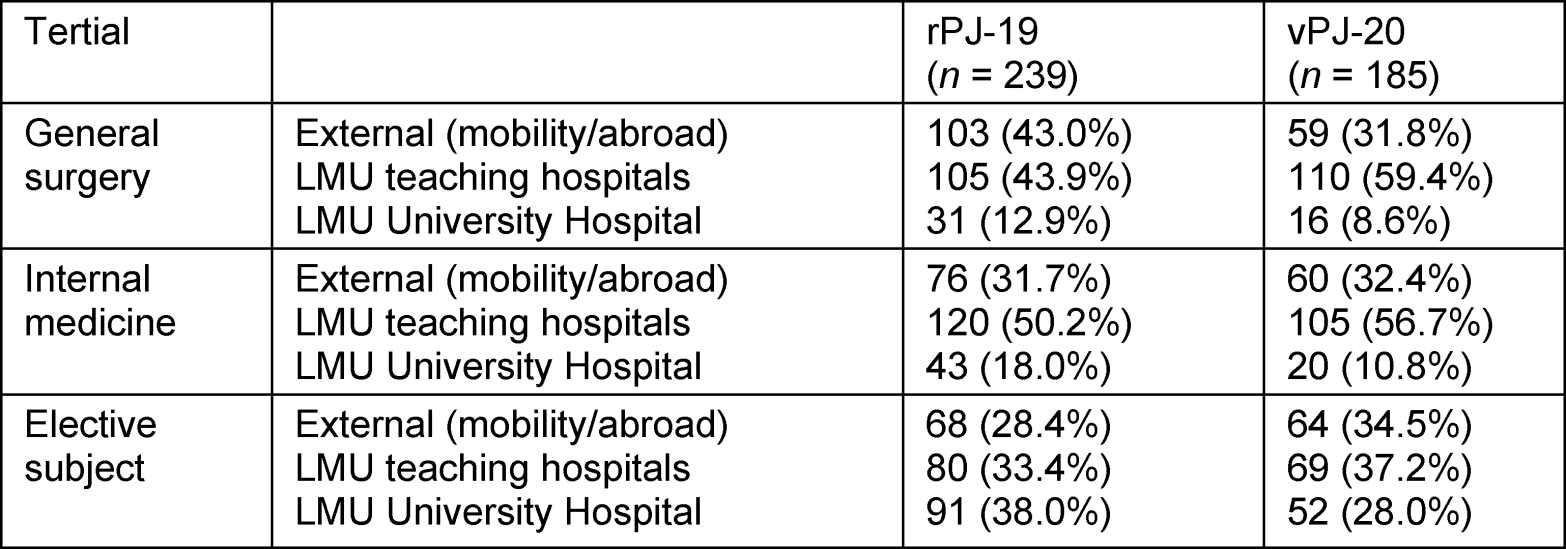
List of PJ locations per subject
