# Supplementary material for: Objective and subjective consequences of pandemic-related study program changes for the perception of the practical year – a comparison of medical students in Germany with and without a second national board examination
Source: GMS J Med Educ. 2023 Jun 15;40(4):Doc53. doi: 10.3205/zma001635 (PMC10407588; doi:10.3205/zma001635)
Supplement: Questionnaire PJ [file JME-40-53-s-001.pdf]

Attachment 1: Questionnaire PJ

Anhang 1 zu Gisi M, Ferrari V, Dubon F, Fischer MR, Angstwurm M, Berndt M. *Objective and subjective consequences of pandemic-related study program changes for the perceptions of the practical year - A comparison of medical students in Germany with and without the second state examination.* GMS J Med Educ. 2023;40(4):Doc53. DOI: 10.3205/zma001635

Questionnaire

1. General information about you

1.1 Age ☐

1.2 Gender ☐

1.3 PJ start ☐

2. What criteria did you use to select your tertials?

If you select more than one answer, please prioritize your response.

		1.	2.	3.	4.	5.	6.	7.	8.	9.	10.
2.1	Good location or city	☐	☐	☐	☐	☐	☐	☐	☐	☐	☐
	Compensation	☐	☐	☐	☐	☐	☐	☐	☐	☐	☐
	Future career (specialty training)	☐	☐	☐	☐	☐	☐	☐	☐	☐	☐
	Recommendations from friends or reviews	☐	☐	☐	☐	☐	☐	☐	☐	☐	☐
	Hospital reputation	☐	☐	☐	☐	☐	☐	☐	☐	☐	☐
	I had a clinical elective at this teaching hospital.	☐	☐	☐	☐	☐	☐	☐	☐	☐	☐
	I already knew the senior physician there.	☐	☐	☐	☐	☐	☐	☐	☐	☐	☐
	Good working atmosphere	☐	☐	☐	☐	☐	☐	☐	☐	☐	☐
	Good PJ teaching	☐	☐	☐	☐	☐	☐	☐	☐	☐	☐
	Working conditions (e.g. working hours)	☐	☐	☐	☐	☐	☐	☐	☐	☐	☐

2.2 Other

☐

3. Tertial planning

3.1 Did you complete your elective tertial in a different elective subject than you planned? ☐ yes ☐ no

3.2 If yes, why?

☐

3.3 Did you complete a tertial at a different teaching hospital than you planned? ☐ yes ☐ no

3.4 If yes, why?

☐

3.5 Have you spent a tertial or half of a tertial abroad? ☐ yes ☐ no

3.6 If yes, why?

☐

3.7 Have you cancelled your tertial or half of a tertial planned abroad? ☐ yes ☐ no

3.8 If yes, why?

☐

3.9 Are you satisfied with the process and organization of your PJ?

☐

4. Future career

4.1 In which specialty do you plan to do your residency?

- ☐ Internal medicine
- ☐ Elective subject
- ☐ General surgery
- ☐ I don't know yet

4.2 Did the PJ influence your decision? ☐ yes ☐ no

4.3 If yes, how?

☐

4.4 Will you apply to your PJ position? ☐ yes ☐ no

4.5 Why?

☐

5. Stress and mental health

	**Stress level**	low					high
5.1	How high was your stress level?	☐	☐	☐	☐	☐	☐
5.2	Can you generally manage stress well?	☐	☐	☐	☐	☐	☐

5.3 Which aspects of the PJ activities were particularly stressful for you?

- ☐ Work in teaching hospital
- ☐ Weekend shifts
- ☐ Relationship with the supervisor
- ☐ Lack of time
- ☐ Work-Life-Balance

Resilience

Please rate the following statements from 1 (strongly disagree) to 6 (strongly agree)

		1					6
5.4	I am a determined person with many plans and projects.	☐	☐	☐	☐	☐	☐
5.5	I am able to multitask and have a lot of energy.	☐	☐	☐	☐	☐	☐
5.6	I like myself and appreciate my work.	☐	☐	☐	☐	☐	☐
5.7	I am self-confident.	☐	☐	☐	☐	☐	☐

Test anxiety

		low					high
5.8	How high do you rate your test anxiety before the M2 exam?	☐	☐	☐	☐	☐	☐
5.9	How high do you rate your exam anxiety before the M3 exam?	☐	☐	☐	☐	☐	☐

Please rate the following statements from 1 (strongly disagree) to 6 (strongly agree)

		1					6
5.10	Through my PJ I feel well prepared for the exam (M2, M3).	☐	☐	☐	☐	☐	☐
5.11	I feel that my PJ has prepared me well for my future work as a physician.	☐	☐	☐	☐	☐	☐
5.12	During my time as a PJ student, I was able to improve my knowledge and skills.	☐	☐	☐	☐	☐	☐
5.13	I was able to prepare sufficiently for the exam (M2, M3) parallel to the PJ.	☐	☐	☐	☐	☐	☐
5.14	During my time as a PJ student, I was able to participate in learning opportunities with sufficient frequency.	☐	☐	☐	☐	☐	☐

6. Corona pandemic

Please rate the following statements from 1 (strongly disagree) to 6 (strongly agree)

		1					6
6.1	I found the restriction of mobility due to the pandemic a real imposition.	☐	☐	☐	☐	☐	☐

6.2	Did you want to complete periods of your PJ abroad and were unable to due to the pandemic?	☐ yes	☐ no
6.3	Have you cancelled any PJ times planned abroad due to the pandemic?	☐ yes	☐ no
6.4	Were you in quarantine or directly affected by consequences of the Corona pandemic (infected, in isolation,...)?	☐ yes	☐ no

6.5 If yes, what?

6.6 How much was the normal PJ routine restricted on your ward?

- ☐ Very much
- ☐ Much
- ☐ A little
- ☐ Not at all

6.7 What restricted the regular PJ procedure on your ward?

- ☐ Short-time work
- ☐ Lack of rotation possibilities
- ☐ Lack of patients

☐ Increased hygiene measures
☐ Reduced participation in rounds
☐ Reduced participation in morning meetings
☐ Reduced participation in courses and lectures
☐ Other reasons

6.8 Other reasons

6.9 What learning content was different because of the pandemic?

6.10 How did you experience the PJ in the Corona pandemic?
☐ Not different than usual
☐ Chaotic
☐ With different content
☐ Other

6.11 Other

6.12 Do you agree with the postponement of the M2 and early PJ due to the Corona pandemic? ☐ yes ☐ no

6.13 Why?

6.14 As a result, did you consider postponing the PJ? ☐ yes ☐ no

6.15 Why?

6.16 Has the postponement of the M2 affected your career planning? ☐ yes ☐ no

6.17 If yes, how?

Please rate the following statements from 1 (strongly disagree) to 6 (strongly agree)

		1					6
6.18	Do you feel disadvantaged by the early PJ?	☐	☐	☐	☐	☐	☐
6.19	Do you feel disadvantaged by the postponement of the M2?	☐	☐	☐	☐	☐	☐

6.20	I feel like I was not heard in the decision on whether to start the PJ early or not.	☐	☐	☐	☐	☐	☐
6.21	I feel like I was not heard in the decision on whether to postpone the M2 or not.	☐	☐	☐	☐	☐	☐

6.22 Free text for comments

